# Supplementary material for: Are carbon emissions trading and green financial instruments synergistic? -Comprehensive quantitative research based on content analysis
Source: PLoS One. 2024 Mar 7;19(3):e0298601. doi: 10.1371/journal.pone.0298601 (PMC10919601; doi:10.1371/journal.pone.0298601)
Supplement: S1 Data — (ZIP) [file pone.0298601.s001.zip › Data/地级市Z_F工作报告.zip!/┴╔─■/┤≤┴1⁄4╩╨/└┤╫╘╣1⁄2╓┌║┼í╛┬φ┐╦╩2╛▌═°í┐_2012.docx]

各位代表：

　　现在，我代表市政府向大会报告工作，请予审议，并请市政协各位委员提出意见。

　　一、2011年政府工作回顾

　　过去的一年，是大连发展历程中极不平凡的一年。我们既收获了经济总量迈上“三个六”新台阶、蝉联“全国文明城市”的喜人成果，也经受了重大安全事故和突发事件的严峻考验。面对复杂多变的形势和前所未有的困难，在党中央、国务院和省委、省政府及市委的正确领导下，市政府团结带领全市人民，以坚定的信心和巨大的勇气，攻坚克难，砥砺奋进，圆满完成了十四届人大四次会议确定的各项任务，实现了“十二五”的良好开局。

　　预计全市完成地区生产总值6100亿元，增长14%；地方财政一般预算收入651亿元，增长30%；固定资产投资4563亿元（按老口径统计为6500亿元），增长30%；社会消费品零售总额1919亿元，增长17%；实际使用外资110亿美元，增长10%；城镇居民人均可支配收入24300元、农民人均纯收入14150元，分别增长14%、15%；城镇登记失业率2.89%。

　　（一）经济运行质量稳步提高

　　坚持以经济结构战略性调整为主攻方向，推动经济增长向投资、消费、出口协调拉动转变。多措并举力促投资稳定增长，全年规划实施千万元以上大项目1328个，其中亿元以上重大项目708个，投资拉动作用强劲。汽车、家电、文化等消费需求持续旺盛，大商集团成为全国百货业首个销售额突破千亿的企业，成功举办国际服装节和国际啤酒节，全市旅游总收入增长18.2%，消费拉动效应凸显。努力克服减量因素增多的影响，实现外贸出口303.5亿美元、增长16%，出口拉动增长的形势好于预期。

　　坚持转型升级和集约发展取向，加快现代产业聚集区建设。华晨专用车基地、格劳博机床等一批重大项目开工建设，一重核岛主设备、LNG、一汽节能与新能源客车生产基地、旅顺北车基地一期工程、东北特钢大连基地、中远造船、固特异轮胎等一批项目竣工投产，瓦房店轴承园区进入全国产业集群50强。省重点推进的22个产业集群销售收入增长32%以上，全市规模以上工业增加值增长16.3%。德豪光电、航天软件研发基地、云计算智慧展示中心、5兆瓦级风电机组等一批战略性新兴产业项目取得进展。服务业聚集区和总部经济、楼宇经济成为城区经济的重要支撑。

　　坚持科技创新驱动，提高经济内生增长能力。生态科技创新城启城，我市成为国家现代服务业产业化基地、创新型软件产业基地、科技与金融结合试点城市。全市发明专利申请和授权分别增长48%和34%，甲醇制烯烃项目获中国专利金奖。物联网应用示范等25个重大科技创新项目国内领先。全市高新技术产品增加值增长29.7%。科技创新对经济社会发展的引领和支撑作用日益突出。

　　（二）全域城市化成效显现

　　全面落实全域城市化规划，按照四大组团总体框架，不断优化全域空间布局和功能区格局。主城区服务经济迈向高端化、集约化，钻石海湾、旅顺绿色经济区等重点区域建设步伐加快。普湾新区起步区、金州新区“十大功能园区”、保税区汽车物流城加速建设，为“三年初具规模、五年基本建成”创造了良好开局。长兴岛临港工业区、瓦房店沿海经济区、花园口新材料基地、庄河临港经济区快速发展。

　　大力强化全域基础设施建设，实施了192项重大基础项目建设。胜利路东段、西部通道北段立交桥、皮炮高速公路通车。大连湾疏港高速、庄盖高速主体工程、振连路开发区段完工。城市地铁、大连北站、旅顺南路快轨、国际会议中心、红沿河核电二期等一批续建工程加快推进。南部跨海通道、渤海大道、三道沟净水厂改造等一批项目启动。以普湾新区为核心、连接主城区和黄渤海区域的重大项目建设全面铺开，全市一体化、网络化的基础设施体系逐步形成。

　　深入推进工业化、城镇化和农业现代化，县域经济实力晋位争先。19个沿海经济带重点园区引进外资占全市的82%，成为开放型经济发展的重要载体。都市型现代农业十大工程全面完成，新建标准日光温室设施农业小区1401个，新增规模以上农业龙头企业40家，粮食和主要农产品产量创历史新高。新农村“三新”工程扎实推进，在256个村实施“六化”工程，31个村实施了整村扶贫项目开发。北三市在全国百强县中的排位大幅前移，其中瓦房店市跃居第13位。县域经济的支撑和拉动作用更加强劲。

　　（三）“三个中心”建设步伐加快

　　加快大窑湾、太平湾、栗子房等12大港区建设，“三核两翼”现代港口集群格局已经形成。长兴岛港获准对外开放。机场三期投入使用，新机场选址及填海工程启动，大连航空公司成立，机场旅客吞吐量超过1200万人次。

以内陆干港、铁海联运体系为支撑的物流中心建设提速，香炉礁物流运输中心主体竣工，空港国际物流园开工建设，金融、信息、物流相融合的大连e港开通。港口集装箱吞吐量完成640万标箱，为实现三年超千万迈出坚实一步。

　　新开业内外资金融机构15家，新增信贷余额和多渠道融资2250亿元，跨境贸易人民币结算居东北之首。全球首个焦炭期货在大商所上市交易，星海湾金融城形成500亿元资金汇集能力。我市金融中心竞争力升至全国第8位。

　　（四）民生质量进一步提升

　　把改善民生作为一切工作的出发点和落脚点，全力推进民生事业发展，市政府为民办15件实事圆满完成，一些事关人民群众切身利益的热点难点问题得到有效解决。

　　实现城镇就业18.9万人，在全国“创业型城市”绩效评估中名列第一。高新区成为“全国模范劳动关系和谐工业园区”。

　　社会保障体系进一步健全，征地和拆迁补偿、农转居保障机制不断完善。实施事业单位绩效工资制度，最低工资、企业退休人员养老金标准再度提高。社会救助体系、老龄和残疾人服务体系建设加快，投入2.6亿元建成城乡社会养老中心85所，我市成为“全国残疾人工作示范城市”。“健康家庭促进计划”惠及38.2万人。实施新农保制度，实现职工医疗保险市级统筹和医保一卡通。

　　建设公共租赁住房3.5万套，廉租住房实现应保尽保，整治弃管楼院29.6万平方米，住房公积金惠及面不断扩大。

　　（五）社会事业全面发展

　　实施教育改革和发展十大工程，完成37所公办幼儿园建设，对613所普惠性公办幼儿园运行给予补贴，中小学公用经费标准大幅提高，区域间教育对口帮扶成效显著，我市成为国家职业教育综合改革试验区。

　　深入推进公立医院和基层医疗卫生机构体制改革，基本药物制度实施范围扩大，新农合最低筹资标准提高到230元。加快新卫生中心等一批重点卫生设施建设。我市及瓦房店市通过“国家卫生城”复审。

　　新科技馆、博物馆、图书馆奠基，市广播电台、电视台完成整合重组，歌舞团、话剧团、杂技团改制基本完成。艺术创作与演出繁荣发展，文化产业不断壮大。市体育中心建设加快推进，市民健身中心完工，我市在全国第七届城运会上摘得奖牌36枚，阿尔滨足球队冲超成功，实德女足夺得全国锦标赛冠军。

　　加强和创新社会管理。完成第8届城市社区居委会直选换届，基层政权建设和民主自治能力得到加强。整合人口、就业、社保等管理职能和服务资源，大力培育新社会组织，推动政府行政管理与群众自治衔接互动。坚持领导接访和包案制度，发挥人民调解组织作用，建立群众诉求表达、利益协调、矛盾调处和权益保障机制，法律援助纳入社会保障体系，群众来信来访呈逐步减少态势。我市成为“五五”普法先进城市。

　　抓好公安“三项建设”，“清网行动”荣立集体一等功。强化安全生产监管，长兴岛和23个乡镇、街道成为“全国安全社区”。加强食品药品安全风险预警和管理，严厉打击制假售假活动。健全突发事件应急体制，创新区域联动、军地联动等应急机制，危机管理和抗风险能力进一步提高。

　　（六）城市管理和环境建设得到加强

　　针对我市处于大项目建设高峰期的实际，大力开展“城市交通综合整治年” 和市容环境治理活动。加快主城区路网建设，通过建管结合解决行车难、停车难问题。加大道路交通执法力度，严厉打击非法营运活动。完善城市管理长效机制，实行行政执法市区街垂直管理，实施环卫事业社会化改革，启动数字化城管系统建设工程。

　　全社会投入100亿元，造林119万亩，城市绿化覆盖率达到45.1%，我市荣获“国家森林城市”称号。

　　坚持低碳、绿色发展，强化资源节约与生态环境保护。加快污水处理设施建设，城市污水集中处理率达90%以上。推动节能与新能源汽车示范推广，901辆节能与新能源汽车投入运行，更新公交客车512辆。实施十大节能降耗工程，全面完成淘汰落后产能任务，万元GDP综合能耗降低3%以上，主要污染物减排2%。大力发展循环经济，大连生态工业示范园区成为国家“城市矿产”示范基地，我市被国家列为餐厨废弃物资源化利用和无害化处理试点城市，被评为中国十大低碳城市第一名。

　　（七）改革开放取得新进展

　　积极推进重点领域和关键环节改革，新市区管理体制改革全面深化，甘井子区综合配套改革稳步推进，省重点发展和支持区域“小政府、大服务”的管理模式初步建立。完成大显集团、棒棰岛食品集团改制和机场集团改建。国有企业直接融资能力明显增强，完成大橡塑股份定向增发，实现重工·起重集团整体上市，市属国有企业资本证券化率达44%。市农村商业银行批筹，首家股权投资引导基金及其管理公司、财务公司成立。农村集体产权制度改革日益深化，社会信用体系建设不断加强。

　　加快沿海经济带开发开放，启动对外开放“大项目年”活动，实施“双百强招商工程”，积极吸引世界500强、产业100强投资，全市新批外商投资企业365家、超千万美元项目24个，实际使用外资连续三年居副省级城市之首。全国首家软件服务外包产业海关保税中心成立。抢抓国内产业转移和南资北上的契机，加大对内招商力度，引进内资1550亿元。第5届夏季达沃斯年会在我市成功举办。大连正在成为沿海地区新一轮开发开放的高地和国内外企业投资兴业的热土。

　　坚定不移支持民营企业转型升级，促进民营经济发展质量、效益和水平不断提升。面对银根紧缩和外部市场需求减弱等困难，千方百计帮助民营企业解决融资难和市场渠道不畅等问题，组织147家担保机构为中小企业提供担保融资283.3亿元，设立54家小额贷款公司为企业投放贷款84.5亿元。通过企业对接、经贸洽谈等形式帮助民营企业拓展国内外市场。预计民营经济实现增加值3600亿元、增长23%。民营企业在税收、就业等方面的贡献率不断上升。

　　（八）政府自身建设不断强化

　　扎实推进学习型政府建设，在“大讨论”活动中凝聚智慧、形成共识，将广大干部群众的真知灼见转化为转变发展方式、破解发展难题的政策措施，不断提高科学决策、依法行政水平，进一步增强驾驭复杂局面和解决实际问题的能力。

　　加快转变政府职能，行政服务效能进一步提高。市区两级行政服务体制机制实现统一联动，网上审批系统对接运行。行政权力运行、工程建设等重点领域的政府信息公开取得突破。公共企事业单位45大类办事公开实现“一网式”发布。切实纠正不正之风，着力解决损害群众利益的突出问题。充分发挥监察、审计等部门职能，从源头上预防腐败，优化发展环境。

　　坚持重大事项向人大报告和向政协通报制度，自觉接受人大及其常委会的法律监督、工作监督和政协的民主监督。认真听取民主党派、工商联、无党派人士的意见和建议，主动接受人民群众和新闻媒体的监督。全年共办理人大代表建议663件、政协提案628件，满意率99.9%。

　　民族、宗教、外事、侨务、对台、气象、人防、防震减灾、档案、双拥共建、民兵预备役等工作都取得了新成绩。

　　各位代表，2011年令人难忘。在各种挑战面前，我们充分依靠全市人民的信任和支持，始终坚持加快发展不懈怠，满怀信心，奋力拼搏，推动经济发展迈上新台阶；始终坚持抢抓机遇不放松，与时俱进，开拓创新，促进城市转型发展迈出新步伐；始终坚持惠民利民不停步，倾心尽力，扎实工作，确保人民群众利益得到新发展；始终坚持直面困难不气馁，风雨同舟，共克时艰，形成凝心聚力建设幸福美好家园的新局面。

　　各位代表，成绩来之不易!这一切得益于党中央、国务院和省委、省政府的正确领导，得益于市委的科学决策，得益于社会各界的关心、支持和帮助，凝聚着全市670万人民的智慧和汗水。在此，我代表市政府，向全市人民，向大力支持政府工作的人大代表、政协委员，向各民主党派、工商联、人民团体和无党派人士，向离退休老同志，向中省直各部门和兄弟省市驻连机构，向驻连部队和武警官兵，向关心支持大连建设发展的海内外朋友们，表示衷心的感谢！

　　在肯定成绩的同时，我们要清醒地看到，我市的发展还存在诸多困难和问题：经济结构调整和发展方式转变任务艰巨；安全生产需要加强、社会稳定工作压力增大；城市环境、社会管理亟须改善；交通、住房、社会保障、收入分配等人民群众高度关注的问题亟待解决；政府服务和发展软环境还需要进一步优化等。

　　二、2012年政府工作目标与任务

　　今年是本届政府届满之年，也是实施“十二五”规划承上启下的重要一年。我市经济社会发展面临的形势十分复杂严峻，世界经济下行风险明显加大，国内经济发展中不平衡、不协调、不可持续的矛盾仍很突出，我市发展受资源、能源、土地和环境等约束加剧。同时，国际环境“危”中有“机”，国内需求潜力巨大，大连仍处在加快发展和转型发展的重要战略机遇期。随着全域城市化的快速推进，我市体制机制创新的活力进一步迸发，综合竞争优势正在提升，经济发展空间逐步扩大。

　　当前我们遇到的问题，都是发展中的问题。我们必须坚持发展第一要务，不被发展中的问题所困扰，不为前进中的曲折所动摇，处理好发展与环境、发展与民生、发展与稳定的关系，进一步坚定科学发展、跨越发展的信心和决心。我们要坚持转变发展方式不动摇，按照依托增量扩大总量、依靠创新优化存量的要求，充分利用临海、临港优势，大力发展和提升石化、装备制造、造船等临港产业，做强做大战略性新兴产业，加快发展现代服务业，不断强化实体经济支撑作用；坚持全域城市化不动摇，高水平推进各城市组团发展，深入推进行政管理体制改革，打破行政区划界限，优化资源配置，创造政策优势，加快沿海经济带各重点产业园区开发建设，形成港口、经济区与城市联动发展新格局；坚持持续改善民生不动摇，把保障和改善民生放在更加突出的位置，集中解决人民群众关心的切身利益问题，多办实事、好事，切实让人民群众共享改革发展成果。

　　2012年政府工作的总体要求是：认真贯彻落实党的十七届六中全会、中央经济工作会议和省、市委十一届二次全会精神，以科学发展观为统领，坚持“稳中求进、稳中求快”，深入实施开放引领、转型发展、民生优先、品质立市战略，大力推进全域城市化，加快“三个中心、一个聚集区”建设，着力转变经济发展方式，继续深化改革开放，切实保障和改善民生，不断加强和创新社会管理，积极推动文化繁荣发展，为建设富庶美丽文明的现代化国际城市而努力奋斗。

　　今年全市经济社会发展的主要预期目标是：地区生产总值增长13%，地方财政一般预算收入增长15%，固定资产投资增长20%，社会消费品零售总额增长16%，实际使用外资增长10%以上，城镇居民人均可支配收入、农民人均纯收入均增长13%，城镇登记失业率控制在3%以内。

　　（一）深化经济结构战略调整，加快转变发展方式

　　以投资结构优化带动产业结构优化，提高生产性投资的经济效益和公益性投资的社会效益，扩大民间投资的范围和领域。全市安排千万元以上项目1841个，投资总额1.62万亿元，年度计划投资3700亿元。落实推进重点项目责任制、月报制和“绿色通道”制度，加强调度、协调和监督，确保在建和续建项目按计划推进，保持投资合理增长。

　　大力发展即期消费、开发新兴消费、挖掘潜在消费。以大型骨干市场和现代商品市场建设为重点，带动社区便民市场和农村中心镇现代商业建设，加快构筑覆盖城乡的便民服务网络。研究制定促进政策，积极引导健康、文化、教育、旅游、养老、环保等消费。加大旧城区、棚户区改造力度，保持房地产业持续健康发展。

　　加大政策扶持和市场开拓力度，实行“一企一策”支持大型出口企业发展，推进出口基地建设。优化外贸结构，扩大机电产品、高新技术产品出口，保持外贸出口稳定增长。

　　根据国家石化产业布局和我市“十二五”规划，瞄准国际先进水平，在构建安全管理体系的前提下，全力推进西中岛绿色石化产业园区规划建设，加快实施大石化(搬迁)、恒力石化、实德百万吨乙烯、延长大型石化等重大项目，推进福佳大化等大孤山半岛石化项目科学有序搬迁，促进石化产业集聚发展。深入实施工业“五项工程”，加快大连湾、旅顺、长兴岛三大造船及海洋工程、金州新区国家数控机床、瓦房店轴承研发制造和旅顺北车基地建设，全面推进工业产业集群跨越发展。

　　积极培育战略性新兴产业，加快高新区世界级软件和信息技术服务基地、花园口国家新材料产业基地建设，推动集成电路、云计算和网络产业发展，促进新能源汽车、储能电池、LED照明等新产品尽快进入市场，全面开展“无线城市”建设。编制完善大连市绿色产业发展规划，加快建设旅顺绿色经济区、庄河国家生态工业示范园区。

　　科学规划海洋资源开发，培育海洋优势产业，加快建设长山群岛旅游避暑度假区和现代海洋牧场，壮大海洋经济。

　　依托城区综合优势，大力发展金融商贸、软件信息、科技服务、会议展览等现代服务业，优先发展楼宇经济、总部经济、网络经济。钻石海湾、新机场沿岸商务区、西城国际商务区、小窑湾国际商务区、金渤海岸现代服务业发展区、恒隆广场等重点项目，要在年内实现更大更快的发展。推进市区“北方不夜港”工程，加快全域温泉旅游发展，办好城市节庆活动，努力把大连建设成为门户型旅游目的地城市。

　　以国家创新型城市建设为引领，启动大连市创造力产业发展示范工程，推动经济发展向内生增长、创新驱动方向转变。着力实施重大自主创新专项，构建10个产业技术创新联盟，建设国家工程技术研究中心和重点实验室等一批创新载体。开展高价值核心专利创造与转化试点，掌握100项核心知识产权，推进10大自主知识产权成果在本地转化。加快生态科技创新城、中科院纳米技术园区、大连（旅顺）科技创新园和科技孵化网络建设，提升数控机床、新能源等国家高新技术产业化基地的集聚效应。

　　（二）加速全域城市化进程，推动区域协调发展

　　全域城市化是落实辽宁沿海经济带开发开放战略的创新之举，是推动全市新一轮发展的强力引擎，也是保增长、调结构、稳物价、惠民生的务实之策，我们一定要解放思想，坚定信心，科学谋划，全力推进。

　　坚持重大基础设施向北部推进、重要资源要素向县域聚集、重点支持政策向农村覆盖，强化各重点区域的城市经济功能，增强生产要素的聚集能力。加快19个沿海经济带重点园区建设，推进基础设施配套，明确主导产业，创新管理体制，完善综合服务功能。加快建设万达金石文化旅游度假区、东风日产整车基地、华晨专用车基地、碳纤维及复合材料产业化基地等分布在各园区的重大产业项目，确保奇瑞汽车、曙光汽车、东北特钢等项目年内达产，尽快形成高端产业竞相聚集、各个区域联动发展的良好局面。继续做好争取国家批准建设长兴岛自由贸易园区工作，支持花园口经济区、旅顺开发区申报国家级开发区，积极申报和争取新市区成为国家综合配套改革试验区。

　　整体规划，加大投入，强力推动一体化、网络化基础设施体系建设。完成大连北站、国际会议中心、振连路、庄盖高速公路项目，推进城市地铁、大连湾跨海交通、渤海大道、长山大桥、公共资源交易市场、金州至普湾城际铁路、南部跨海通道、新机场、大伙房输水入连、长海县跨海引水等项目建设，切实增强各区域的城市功能和承载能力。

　　加快县域重点园区和中心城镇开发建设，培育特色主导产业和工业产业集群，推动县域经济跃上新台阶。大力发展都市型现代农业，加快农业科技创新，推进农业集约化、标准化和产业化经营，发展设施农业10万亩，新增规模以上农业龙头企业30家。创建庄河国家现代农业示范区和20个市级现代农业示范区。推进新社区、新村庄、新农庄建设，在150个村实施“六化”工程，实施43个低收入村整村扶贫开发。

　　（三）推进“三个中心”建设，提升城市核心功能

　　优化“三核两翼”港口布局规划，完善大窑湾核心港区功能，加快太平湾、栗子房等航运中心关键节点以及旅顺新港等港口群建设，促进区域港口整合，探索多元化投资和经营之路。推进大窑湾北岸开发、长兴岛专业码头等工程，力争完成大连湾疏港高速公路、新机场填海主体工程，加快长海机场扩建。探索建设以保税港区和大连e港为依托的高端港航服务业体系。

　　完善东北亚国际物流中心发展规划，加快构建面向东北腹地及环渤海地区、辐射东北亚区域的国际物流网络体系。打好大连港集装箱吞吐量超千万关键之年攻坚战，集装箱吞吐量增长25%。加快内陆干港布局建设，发展保税物流，搞好铁海联运示范项目建设。推进大连空港国际物流园、香炉礁物流园等重点物流园区建设，加快货物集散中心向资源配置中心转变。进一步改善通关环境。

　　深入实施金融中心建设规划，发挥期货市场龙头作用，支持大商所实施品种、人才和国际化战略，建设亚洲重要的期货中心。推进人民路、星海湾等金融功能区建设和发展，提升金融机构聚集度。加快金融改革创新步伐，推动多层次资本市场和股权投资业发展，完善农村金融、中小企业金融服务体系，探索航运金融、科技金融、绿色金融等领域的创新。扩大信贷投放和多渠道融资，新增贷款和融资不低于2300亿元。

　　（四）切实保障和改善民生，不断增加人民福祉

　　进一步稳定和扩大就业，全年实现城镇实名制就业18万人。认真落实生源储备和“三支一扶”计划，重点抓好高校毕业生就业，做好失地失海农民、大龄人员等困难群体的就业服务工作。深入推进创建国家级创业型城市工作，以创业带动就业。大力开展职业培训，全面提升劳动者从业素质。启动人力资源和社会保障档案中心建设。

　　加快完善城乡居民养老保险制度以及失地失海、农转居人员的养老保障政策，有序开展进城务工农民参加养老保险，加快推进机关事业单位失业、工伤、生育保险市级统筹。逐步提高企业退休人员基础养老金和城乡居民社会保险待遇水平，深入推进“金保工程”。

　　着手制定实施方案，规范收入分配秩序，逐步解决区域间收入差距过大问题。落实城乡低保标准自然增长机制，加大城乡困难居民临时救助和低保分类救助力度，完善慈善基金长效救援机制，强化老龄和残疾人保障服务体系，加快市残疾人托养中心、殡仪馆等重点民生项目建设。努力保持物价稳定，抓好“菜篮子”工程，适时启动物价补贴。

　　安排保障性住房2.8万套，廉租住房应保尽保，加大住房公积金对公租房建设的支持力度，完成12万平方米弃管楼院的整治任务。

　　推进义务教育均衡发展，北三市全部达到省“双高”普九标准，继续实施城乡教师能力提升工程，推动高中教育优质特色发展。强化校车安全，开展农村中小学校车运营试点工作并提供补贴。新建、回收20所公办幼儿园，新开办普惠性幼儿园50所，改善农村幼儿园设施条件。加快职业教育基地建设和资源整合，组建装备制造业等3大领域职教集团。推进在连高校与城市互动发展。

　　加强城市社区和农村基层医疗机构建设，有效解决就近看病和医疗资源合理利用问题，完善城市大医院与基层医疗机构对口支援协作机制，多措并举提高医疗卫生服务能力和技术水平。推进市儿童医院、卫生中心等医疗设施建设。新农合最低筹资标准提高到320元，在涉农地区开展免费孕前优生健康检查。深入开展爱国卫生运动，推进健康城市建设。

　　（五）加强和创新社会管理，维护社会和谐稳定

　　完善党委领导、政府负责、社会协同、公众参与的社会管理格局，加强法律、制度建设和基层服务，努力解决影响社会和谐稳定的突出问题，维护人民群众权益，促进社会公平正义，保持社会良好秩序。开展“六五”普法活动，提升法治城市建设水平。从企业信用入手，全面推进信用体系建设。

　　深化街道管理体制改革，按照管理、服务、执法三位一体要求，理顺管理服务机制，实现工作重心向社会管理和公共服务转移。推动 “两新”组织发展，注重发挥各类行业协会、商会的作用，形成层级服务管理和分类监管网络。

　　健全人口社会管理机制，加强流动人口服务管理，完善出租房屋信息申报登记制度，帮助流动人口解决就业、就学、就医、社会保障等基本生活问题。建立计生奖励扶助政策动态调整机制。

　　创新工作载体，提高社会稳定工作能力。实施“和谐社区”工程，把社区建成依法自治、管理有序、服务完善、文明和谐的社会生活共同体。实施“平安大连”工程，健全人防物防技防相结合的现代化、立体化社会治安防控体系。实施“亲民维稳”工程，完善群众诉求表达、利益协调、矛盾调处和权益保障机制。实施“舆情疏导”工程，使互联网等公共媒体成为传播先进文化、通达社情民意、化解社会矛盾的重要渠道。

　　完善应急管控体系和联动机制，形成城乡救援一体化模式，做好重点应急物资装备保障，提高处置公共安全危机和突发事件的能力。完善食品安全监管体系，加强日常监管与专项整治，在旅顺规划建设大连绿色食品工业园区。严格落实安全生产企业主体责任、部门监管责任和属地管理责任，切实强化安全生产综合监管和防范体系建设，持续深入开展危险化学品、矿山、建筑、交通运输、渔业、消防等重点行业和领域安全隐患排查治理，坚决遏制重大安全事故发生。

　　（六）推动文化大发展大繁荣，努力建设文化强市

　　制定出台建设文化强市实施意见，把文化建设放在全局工作的重要战略地位，创新发展理念，推进体制改革，促进文化事业、文化产业同步发展，增强城市文化软实力。

　　以创建全国公共文化服务体系示范区为抓手，提高公共文化产品和服务的供给能力。新建大连群众艺术馆，完善公共图书馆、文化馆、博物馆、影剧院等设施功能。实施太阳沟、胜利桥北等历史文化街区改造。建立四级公共文化服务网络，开展“大连读书月”活动和送戏下乡、农村电影“2151”工程，进一步丰富基层群众文化生活。扶持京剧院建设国家一流院团，支持杂技团建设大马戏团，推动文学、戏剧、影视等领域创作出更多的精品力作。

　　全面落实文化产业“十二五”发展规划，重点推进建设产业基地、打造知名品牌、培育龙头企业、加强人才集聚、营造产业环境五项工程，实现文化产业增加值年均增长20%以上，逐步使文化产业成为我市的支柱产业。推动文化产业集群发展，搞好大连文化产业创新园区等重点项目规划建设，支持一大批文化骨干企业发展壮大，引导和鼓励社会资本以多种形式参与重大文化产业项目和文化产业园区建设。

　　深化国有文化单位改革，完成经营性文化事业单位转企工作，推进新闻出版体制改革和公益性文化事业单位改革。着力构建统一、开放、竞争、有序的现代文化市场体系，创新文化市场管理体制，营造“文化安全”的市场秩序。完善政策保障机制，加大财政对文化事业的投入，强化在用地、金融、税收等方面对文化产业的扶持。推进文化对外交流合作。

　　全力做好“十二运”筹备工作，加快市体育中心、普湾新区体育中心、瓦房店帆船帆板基地和金州激流回旋等比赛场馆建设及周边环境整治，加强体育人才培养，努力提高竞技水平。支持大连足球再创佳绩。

　　（七）深入推进改革开放，增强创新发展动力

　　坚持新市区改革方向，加快理顺体制、机制，推进功能区与行政区深度融合。深化市级综合配套改革试点，加大事权下放和政策支持力度，强化试点区体制优势和引领作用。优化公共财政支出结构，重点保证在建续建项目、社会民生事业、全域城市化基础设施建设的资金需求。

　　深化国有企业改革，健全法人治理结构。强化国有企业资产重组和资本运营，加大国企上市和海内外并购力度，提高国企直接融资能力，形成以大连装备制造投资有限公司为龙头的国企投融资体系，推进热电集团整体上市。支持国有公益性企业改革发展。完成事业单位岗位设置管理工作，健全以聘用合同管理为基础的用人机制。

　　推进农村改革，完善土地承包经营权流转机制，发展多种形式的适度规模经营，促进农业生产经营模式创新。加快县、乡两级林权流转管理服务中心建设。推进城乡建设用地增减挂钩，促进节约集约用地。

　　继续扩大利用外资，大力引进重大工业地产、旅游地产、商业地产和先进制造业、战略性新兴产业、现代服务业项目。创新利用外资方式，扩大境外上市、发债、国际基金投资，吸引国际知名企业在连设立研发中心、地区总部、生产基地、采购中心和服务外包基地，鼓励外商增资扩股。注重引进先进技术、管理理念和高端人才，办好“海创周”和“海创工程”。加快“走出去”步伐，支持优势企业采取多种形式扩大境外投资、承包境外工程项目。

　　加强对内招商引资，继续将长三角、珠三角地区作为主战场，充分发挥区市县、重点园区和企业的主体作用，拓展区域合作领域，提高交流合作水平，引进内资增长20%以上。

　　进一步完善服务体系和政策措施，拓展民营经济发展空间。实施中小企业融资促进行动，综合运用信贷、保险、基金、上市融资、股权投资等融资工具，有效引导金融资金、社会资金加大对中小企业的投入。坚持一视同仁，着力加强和改善对中小企业用地、立项、监管、产品销售、市场开拓的政策支持和管理服务。积极推动中小企业创新转型发展，形成“专精特新”竞相发展的良好格局。

　　（八）加快绿色大连建设，再造宜居城市新优势

　　巩固森林城市建设成果，实施森林乡村、湿地公园、生态河、海防林等青山生态系统十大工程。全社会再投入100亿元，造林90万亩，植树2亿株，提高造林质量，城市绿化覆盖率达到45.2%。加快公园及游园绿地改造，提升主干路及重点区域绿化品质。

　　继续实施城市交通综合整治，努力解决交通拥堵和停车难问题，优先发展公共交通，营造安全、便捷、舒适的交通环境。推进“七横七纵”快速路网建设，实施川岭路、中华路改建工程。继续抓好城建“五个一、三个一”工程，精细化维修城区道路，新建农村公路300公里。

　　提高城市常态化管理水平，推进中心城区数字化管理，加快“智能城市”建设。坚持建设与管理并重方针，持续开展市容环境综合整治，倡导文明施工，控制工程扬尘和沙石撒漏，保持在建工程现场及周边地区环境清洁有序，抓好街巷路和老旧小区环境卫生改造，解决私搭乱建、侵占绿地、占道经营问题，加强城市养犬、广告管理。完善和提升城市污水处理设施建设，推进水务现代化建设试点。

　　切实加强节能减排，完善工业企业能源审计、能效对标、能耗限额管理制度，加强节能管理、监察和服务体系建设。积极应用新型能源，坚决淘汰落后产能，全市万元GDP综合能耗降低3.8%左右。加快建设主要污染物减排重点项目，推进重点化工园区在线监测系统建设，启动市区PM2.5和臭氧监测。科学保护海洋环境。

　　大力发展循环经济，加快构建农业生态系统网络和工业循环经济产业体系，重点发展再制造产业、生态旅游、绿色餐饮住宿和循环物流业，加快再生资源利用产业整合和集中布局。

　　继续做好民族、宗教、外事、侨务、对台、气象、人防、防震减灾、档案、双拥共建以及民兵预备役等工作。

　　各位代表！面对复杂的形势和艰巨的任务，各级政府和全体公务员必须不断加强自身建设，努力提高驾驭复杂形势、推动科学发展的能力和水平。

　　我们一定要不畏艰难，勇于承担，满怀发展激情，坚定必胜信心，以良好的精神状态、创新的工作举措、扎实的工作作风，去化解各种困难和风险，抓好各项任务落实，努力开创振兴发展的新局面，推动我市经济社会平稳较快发展。

　　我们一定要坚持依法行政，提高依法科学民主决策水平，自觉接受人大法律监督、工作监督和政协民主监督，认真落实人大代表议案，做好人大代表建议和政协提案办理工作，规范决策程序，全面建设法治政府。

　　我们一定要下大力气改善软环境，通过开展“软环境建设年”活动，坚决纠正吃拿卡要等影响经济社会发展的突出问题，营造良好的“安企、保商”环境。抓好“五大系统”建设，加强对重大专项资金和领导干部的经济责任审计，着力治理腐败易发领域存在的问题，不断提高政务服务水平。

　　我们一定要加快转变政府职能，强化政府管理创新和公共服务。规范行政审批程序，推进网上审批。拓展政务公开的广度和深度，推进电子政务信息资源共享，加强机关质量标准管理，着力提高行政效能。

　　我们一定要切实转变工作作风，坚持说实话、办实事、求实效，把精力放在抓事业上，把本事用在抓发展上，把能力和水平体现在抓成效上，坚持埋头苦干，务求干则必成，努力创造一流的工作业绩。

　　各位代表！展望新的一年，我们激情满怀、信心百倍。让我们更加紧密地团结在以胡锦涛同志为总书记的党中央周围，在中共大连市委的领导下，振奋精神，顽强拼搏，开拓进取，扎实工作，努力完成本届政府各项工作任务，以优异的成绩迎接党的十八大胜利召开！
